# Advances in Surface-Enhanced Raman Spectroscopy for Therapeutic Drug Monitoring

**DOI:** 10.3390/molecules30010015

**Published:** 2024-12-24

**Authors:** Huasheng Lai, Xinlan Wang, Menghan Qi, Hao Huang, Bingqiong Yu

**Affiliations:** Jiangxi Province Key Laboratory of Pharmacology of Traditional Chinese Medicine, School of Pharmacy, Gannan Medical University, Ganzhou 341000, China; wangxinlan@gmu.edu.cn (X.W.); qimenghan@gmu.cn (M.Q.); hhuang@gmu.edu.cn (H.H.)

**Keywords:** surface-enhanced Raman spectroscopy, therapeutic drug monitoring, enhancement substrate, analytical strategies

## Abstract

Therapeutic drug monitoring (TDM) is pivotal for optimizing drug dosage regimens in individual patients, particularly for drugs with a narrow therapeutic index. Surface-enhanced Raman spectroscopy (SERS) has shown great potential in TDM due to high sensitivity, non-destructive analysis, specific fingerprint spectrum, low sample consumption, simple operation, and low ongoing costs. Due to the rapid development of SERS for TDM, a review focusing on the analytical method is presented to better understand the trends. This review examines the latest advancements in SERS substrates and their applications in TDM, highlighting the innovations in substrate design that enhance detection sensitivity and selectivity. We discuss the challenges faced by SERS for TDM, such as substrate signal reproducibility and matrix interference from complex biological samples, and explore solutions like digital colloid-enhanced Raman spectroscopy, enrichment detection strategies, microfluidic SERS, tandem instrument technologies, and machine learning-enabled SERS. These advancements address the limitations of traditional SERS applications and improve analytical efficiency in TDM. Finally, conclusions and perspectives on future research directions are presented. The integration of SERS with emerging technologies presents a transformative approach to TDM, with the potential to significantly enhance personalized medicine and improve patient outcomes.

## 1. Introduction

Therapeutic drug monitoring (TDM) is defined as the process of measuring a drug’s concentration in body fluids and interpreting the results to optimize the dosage regimen for individual patients [1,2]. TDM is particularly vital for drugs with a narrow therapeutic index, where the difference between a therapeutic and a toxic concentration is minimal. This precision in dosing is crucial to maximize the drug’s efficacy and minimize the risk of adverse effects [3]. TDM plays multifaceted roles in clinical practice: (i) allows for personalized dosing based on individual pharmacokinetics; (ii) identifies and corrects subtherapeutic or toxic drug levels quickly; (iii) manages drugs with narrow indexes, preventing severe clinical issues; (iv) measures compliance by detecting inconsistencies in drug levels; and (v) guides dosing in special populations, accounting for pharmacokinetic differences [4,5].

TDM depends on analytical methods to measure drug concentrations accurately and reliably in biological matrices such as blood, plasma, serum, and saliva [6,7]. Currently, chromatography and related hyphenated techniques are the gold standards for drug analysis due to their high resolution, sensitivity, specificity, and quantification [8,9,10]. However, these methods suffer from time-consuming sample preparation, complex operation, high initial costs of equipment, and ongoing costs for maintenance. To improve the efficiency of TDM, a rapid, accurate, and efficient analytical method with easy operation and economic cost is required.

Raman spectroscopy, an optical label-free modality, has been used for drug analysis due to its fast capacity to provide molecular vibrational information. However, it has several inherent limitations that make it less suitable for TDM. Raman spectroscopy suffers from inherently weak signal intensity, which requires high laser powers and long integration times for detection [11]. This results in a slow and inefficient analytical process. Furthermore, Raman spectroscopy is susceptible to interference in complex biological matrices, such as blood and plasma, due to the presence of strong fluorescent backgrounds and overlapping spectral features [12]. This susceptibility limits its sensitivity for detecting trace levels of drugs, which is critical for drugs with narrow therapeutic windows where even small variations in concentration can lead to significant clinical outcomes [13].

Surface-enhanced Raman spectroscopy (SERS) is a popular TDM technology that addresses these limitations. SERS offers non-destructive analysis, specific fingerprint spectra, high sensitivity, low sample consumption, simple operation, and low ongoing costs [14,15]. The enhancement of the Raman signal by plasmonic nanostructures in SERS allows for the detection of trace levels of drugs, overcoming the sensitivity limitations of traditional Raman spectroscopy. Additionally, SERS provides specific and distinct spectral fingerprints that can be used to differentiate drugs from the complex biological background, reducing interference and improving selectivity.

Rapid monitoring is critical for TDM, especially for drugs with narrow therapeutic windows or in emergency scenarios like intensive care unit (ICU) settings. Timely adjustment of drug dosages can prevent therapeutic failures and toxicities, which can be life-threatening. SERS enables real-time monitoring of time-dependent drug concentrations and imaging, which is crucial for immediate clinical decision-making [16]. The rapid and accurate detection capabilities of SERS make it superior for TDM applications, particularly in scenarios where swift responses are necessary to ensure patient safety and to optimize therapeutic outcomes.

Despite the advantages of SERS, it also faces challenges such as unsatisfactory selectivity due to indiscriminate hotspot enhancement effects, time-dependent adsorption effects between drug molecules and enhancement substrates, and the complexity of establishing standardized protocols. Ongoing research is focused on improving substrate design, reducing costs, developing user-friendly data analysis tools, and establishing robust standardization protocols to enhance the technique’s utility and acceptance in clinical settings.

**Figure 1:**
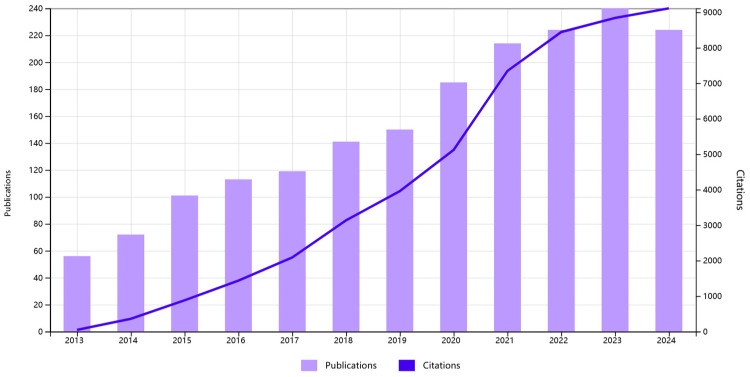
Trends of publications and citations on SERS for TDM, according to the *Web of Science*.

The incorporation of SERS in TDM offers an innovative approach, leveraging its high sensitivity and specificity to accurately measure drug concentrations in biological samples, thus enhancing the precision of personalized medicine. Given the rapid development of the field (Figure 1), this review aims to provide a comprehensive understanding of the entire scenario. Past works have reviewed the development of SERS-TDM in clinical settings and pharmacies; however, few of them have reported strategies for SERS in TDM [1,17,18,19]. Thus, this review focuses on SERS techniques that have been used for TDM in the past few years. First, SERS enhancement substrates that are widely used for TDM are introduced since SERS is highly dependent on substrates for Raman signal enhancement. Then, the application of SERS enhancement substrates and relative analytical methods for TDM under different scenarios are reviewed. Finally, conclusions on SERS for TDM are drawn.

## 2. SERS Substrates for TDM

The enhancement substrate plays a crucial role in SERS application. The design and preparation of SERS substrates are key factors in achieving further development of SERS. By assembling or growing plasmonic metal nanostructures on a substrate, the Raman signal of drug molecules can be significantly enhanced, thereby improving the sensitivity of detection. Based on the different purposes of TDM, different SERS substrates have been developed to enhance signals, improve detection sensitivity, ensure reproducibility and stability, selectively enrich target molecules, and simplify sample pretreatment, as well as for multidimensional surface plasmon coupling and synergistic enhancement. In this section, different states of frequently used SERS substrates for TDM are introduced, including colloidal plasmonic nanoparticles, composite suspensions, and solid substrates.

### 2.1. Colloidal Plasmonic Nanoparticles

Plasmonic nanoparticles are nanoscale particles that exhibit unique optical properties due to their interaction with light, particularly in the visible and near-infrared regions. These properties originate from the collective oscillation of electrons within the metal, known as localized surface plasmon resonance (LSPR) [20]. This phenomenon results in significant enhancement of electromagnetic fields near nanoparticles, with applications in sensing, imaging, and photothermal therapy. Various structured plasmonic nanoparticles have been investigated due to their structure-dependent optical properties. By adjusting the size and shape of nanoparticles, the SPR wavelength can be precisely manipulated, thereby achieving precise manipulation of light [21]. Additionally, plasmonic nanoparticles are capable of penetrating cell membranes in significant quantities and can responsively detect the biological transformation of therapeutic drugs within cells. Currently, colloidal plasmonic nanoparticles used for TDM include gold, silver, and copper nanoparticles with different morphological and structural compositions [22,23,24,25].

Colloidal plasmonic nanoparticles are popular for SERS application in TDM due to their simple preparation, adjustable size and morphology, and the LSPR in the NIR region. Zhao et al. [24] developed a modified Ag colloid as an SERS substrate to achieve ultrasensitive detection of the antitumor drug methotrexate (MTX), with a limit of detection (LOD) of 1360 cm^−1^ (the C=C stretching mode of the pteridine ring) down to 0.1 fmol/L. The salt-induced aggregation of Ag colloid exhibited giant electromagnetic field enhancement and generated more “hot spots”. To improve signal stability for quantitative analysis, 2D nanoparticle arrays assembled at liquid–liquid interfaces were produced [25]. Bell et al. [26] constructed monolayer colloidal plasmonic nanoparticle films by interfacial assembly, which, with good analytical practice, could be used for reliable and quantitative in situ SERS monitoring of drugs. The two-dimensional plasmonic nanoparticle films, which have easy, cheap, and scale-up production ability, exhibited good sensitivity and signal uniformity in quantitative analysis. By adjusting the composition, size, spacing/positioning, configuration, interparticle gaps, and porous properties of metal nanostructures, maximum near-field enhancement can be achieved.

Bimetallic plasmonic nanoparticles were also developed to adjust the spectral window and LSPR and to improve the electromagnetic enhancement of monometal nanoparticles [27]. Zhang et al. [28] developed core-shell Au@Ag NPs for the detection of 6-mercaptopurine and its metabolic product, 6-mercaptopurine ribose, and for real-time imaging of tumor cells. In comparison to the nonporous nano-structures, porous bimetallic nanoparticles have been developed with enhanced adjustability in porosity and composition, larger specific surface areas, and a higher concentration of hotspots. Their plasmonic peaks can be tuned within the biological transparent window in the near-infrared (NIR) region, which is crucial for various applications. The drug delivery efficiency was enhanced, and adsorption capacity increased due to a higher surface area. Meunier et al. [29] prepared hollow porous Au-Ag NPs with rough surfaces and high porosity of 30–60% and improved enhancement of single-particle SERS quantitative monitoring of the release of loaded Doxorubicin drug.

Although colloidal plasmonic nanoparticles are easy to prepare and use, their weak adsorption to therapeutic drugs severely limits their application. A prolonged adsorption process affects the timeliness of drug testing, especially in POCT and ICU. Wang et al. [30] proposed a freeze–thaw–ultrasonication method for preparing colloidal Ag nano-aggregates; the nano-aggregates were then assembled on a SERS detection platform to realize continuous online monitoring of 5-nitro-8-hydroxyquinoline with high-throughput.

### 2.2. Composite Suspension

Composite SERS substrates leverage the advantages of various materials to enhance the intensity, stability, and functionality of SERS signals, which are crucial for biomedical applications. Functional materials and porous materials for preparing Au/Ag composite SERS substrates to improve adsorption, separation, and sensitivity have been introduced [31].

Metal–organic frameworks have ultra-high porosities and large specific surface areas that provide space for attaching many metal nanoparticles to prevent their aggregation while also serving as a solid-phase extraction medium to enrich the target molecules to be tested [32,33]. MOFs are regarded as high-performance adsorbents for improving sensitivity and assisting with the elimination of potential interfering biological substances in the samples during SERS for TDM. Zhan et al. [34] prepared a Au/Ag@ZIF-8 nanocomposite that could be used for solid-phase extraction of tacrolimus from human serum, with an LOD of 6.4 ng/L at 1134 cm^−1^. This LOD is 10^3^–10^6^ times lower than that of reported analytical methods.

Magnetic nanomaterials can be rapidly separated under an external magnetic field, achieving efficient enrichment of target molecules, which is beneficial for the pretreatment of complex samples [35,36]. Modified magnetic nanoparticles can quickly capture, enrich/concentrate, and separate target biomarkers with the help of an external magnetic field and the force of a magnetic trap; they can then be combined with SERS for swift detection. Feng et al. [37] developed a magnetic Fe_3_O_4_@SiO_2_@MIL-101(Fe) SERS substrate for rapid magnetic solid-phase extraction and detection of carbamazepine at 728 cm^−1^ and clozapine at 1054 cm^−1^, with LODs of 0.072 and 0.12 mg/L, respectively.

Two-dimensional materials are popular for biomedical applications due to their big surface areas, excellent biocompatibility, biodegradability, and unique physicochemical properties [38,39,40]. These properties have important applications in biosensors, bioimaging, and photothermal therapy. Two-dimensional materials used for TDM include, but are not limited to, graphene, MXene, and metal hydroxides. Xue et al. [39] introduced Ti_3_C_2_T_x_ MXene nanosheets modified using Ag nanocubes as dual-enhancement SERS substrate for the precise analysis of ritonavir at 1355 cm^−1^ and ibrutinib at 1444 cm^−1^ in serum samples with LODs of 0.562 and 1.17 μg/L. Lai et al. [40] prepared a rare earth metal hydroxide nanosheet and modified it with Au@AgNPs to form a SERS substrate that can be used for rapid adsorption and determination of 6-mercaptopurine in 1 min.

Probe molecules and aptamers are also popular for plasmonic nanoparticle modification to improve selectivity or specificity [41]. Liz-Marzán et al. [42] used a pH-sensitive probe to modify a Au nanostar and then encapsulated it in a layer of biocompatible polymer coating, resulting in a substrate that could realize SERS monitoring of local pH in encapsulated therapeutic cells. Yang et al. [43] modified a porous anodized aluminum substrate with an aptamer; hybridization of another aptamer modified with 4-ATP@AgNPs achieved rapid and selective enrichment of tetracycline in 1 min and sensitive detection in 3 min, with LOD of 1 fg/mL at 1080 cm^−1^.

Additionally, the above-mentioned materials can be used in combination as multi-functional SERS sensors, resulting in improved TDM performance. For example, Zhou et al. [44] prepared Fe_3_O_4_@Au@Ag NPs with graphene oxide (GO) as a nanocomposite SERS substrate and then modified it with a 4-mercaptophenylboronic acid (4-MPBA) molecular probe. 4-MPBA directly interacts with doxorubicin (DOX) through the boronic ester linkages. The GO-Fe_3_O_4_@Au@Ag-MPBA probe realized real-time SERS monitoring of anticancer drug release, SERS/MR imaging, and pH-sensitive chemo-phototherapy. It should be noted that the used substrate includes, but is not limited to, the above materials.

### 2.3. Solid Substrates

Solid SERS substrates exhibit significant advantages over colloidal substrates in terms of reproducibility, stability, integration, and adaptability, making them more promising for practical applications [45]. By precisely controlling the shape and arrangement of nanostructures, solid substrates can achieve higher signal enhancement effects and provide stronger Raman signals [46]. Liz-Marzán et al. [47] presented a three-dimensional network construct of a hydrogel-based scaffold containing AuNRs that could be used to monitor 3D drug diffusion in cell environments using SERS. Panikar et al. [48] prepared gold-film-coated silica nanoparticles on glass as a solid Au@SiNP SERS substrate via self-assembly and sputtering. The Au@SiNP SERS substrate, which is uniform in size and has a periodic structure, generated multiple hotspots at the nanoscale. The zwitterionic layer of modified l-cysteine on gold surfaces lowers serum fouling by 98.5% compared to bare gold surfaces, and the modified l-cysteine Au@SiNP SERS substrate can be utilized for TDM of doxorubicin in undiluted serum samples down to 20 nmol/L.

Hydrogel and polymeric SERS substrates have attracted a lot of attention in TDM due to their good biocompatibility [49]. By adjusting the mesh size of the hydrogel, the target can be selectively screened. Wang et al. [50] prepared Ag@PNIPAM microgels with a three-dimensional reticulated structure, tunable pore sizes, a large surface area, and excellent biocompatibility. The microgels are suitable for solid-phase extraction and SERS determination of aminophylline in human serum, achieving an LOD of 0.61 μg/mL at 572 cm^−1^. Bell et al. [51] developed swellable polymer films using stabilized Au/Ag NPs in dry hydroxyethylcellulose films. The films swell quickly and can rapidly absorb the anticonvulsant drug phenytoin and release AgNPs, facilitating SERS detection at levels as low as 1.8 mg/L.

Solid substrates are easy to integrate with other technologies, such as microfluidic chips, making them suitable for developing portable and high-throughput detection devices. Göksel et al. [52] dropped AgNPs on a three-fork chip with nanopillars on the surface of a microfluidic device to achieve fast nanopillar-assisted separation and SERS quantification of methotrexate in human serum in 10 min, with a limit of quantification of 2.1 μmol/L at 679 cm^−1^.

SERS substrates embedded in highly integrated wearable devices are becoming a popular development direction. Xiao et al. [53] prepared a Au nanocone array on a silicon wafer embedded in PDMS microfluidics and then assembled it in a wearable sweat biosensor that could monitor acetaminophen and metabolism with an LOD of 0.13 μmol/L at 1162 cm^−1^. These highly integrated sensors with noninvasive features and molecular tracking superiority provide us with a new solution for label-free and sensitive POCT.

Compared with liquid substrates, solid substrates are easy to carry and operate, can significantly improve the stability of Raman signals, and expand the practical application range of SERS analysis, especially in reducing the need for sample pretreatment. Additionally, solid-state substrates with concentrated hotspots per unit area can provide high-intensity SERS signals for TDM. However, solid SERS analysis also faces several challenges, including (i) cost and preparation complexity: the high cost of preparation methods, such as magnetron sputtering and nanolithography, limits their commercialization; (ii) long surface modification times: the substrate surface prepared by physical methods is pure, which means that the adsorption of the target drug is hard and surface modification takes a long time; (iii) structural instability: hotspots on SERS substrates may undergo structural changes due to laser irradiation, resulting in changes in the shape, size, and spacing of nanoparticles, affecting the stability of SERS signals; (iv) limited application scope: solid SERS substrates are mainly used for in vitro analysis and are not suitable for in vivo analysis.

## 3. Emerging Strategies for SERS in TDM

SERS has made significant strides in TDM, providing a sensitive and specific method for quantifying drug concentrations in biological samples. The technology addresses key challenges in TDM, such as the need for rapid, accurate, and cost-effective analysis. Innovations in SERS substrate design have improved detection sensitivity and selectivity; however, new approaches for enhancing analytical performance are needed. In this section, emerging advanced SERS analytical technologies for TDM are introduced, including digital colloid-enhanced Raman spectroscopy, enrichment detection, microfluidic SERS, and machine learning. These strategies have been successfully used for the detection of different therapeutic drugs, as shown in Table 1. Most of the detected therapeutic drugs are molecules with strong Raman activity, such as those containing aromatic rings, conjugated systems, or functional groups like thiols, amines, and carboxyl groups, which interact strongly with plasmonic surfaces. Additionally, there are some specific drugs with weak adsorption and interaction with plasmonic surfaces whose detection can be achieved using immunology-based methods.

### 3.1. Digital Colloid-Enhanced Raman Spectroscopy

Colloidal SERS substrates may aggregate or settle during storage and use, resulting in uneven distribution of hotspots, which in turn affects the stability and signal uniformity of SERS detection. The use of colloidal SERS substrates typically requires complex sample preparation steps such as centrifugation, washing, and redispersion, which increases the time and operational difficulty of analysis. Due to the possible changes in the aggregation state and distribution of colloidal particles over time, the generated SERS signal intensity may be inconsistent, making quantitative analysis difficult.

**Figure 2:**
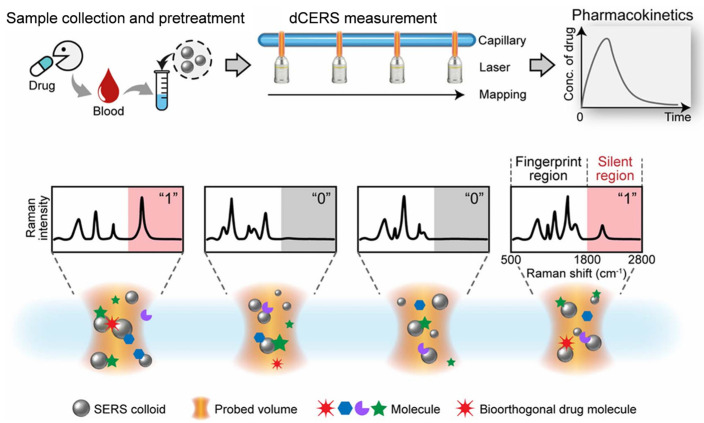
Illustration of the use of dCERS for the pharmacokinetic detection of bioorthogonal drugs; adapted from Ref. [54], reproduced with permission.

The problem of repeatability, which is the main bottleneck hindering the large-scale application of SERS, has not been truly solved since the discovery of the SERS technology 50 years ago. In 2024, Ye et al. [64] reported a digital colloid-enhanced Raman spectroscopy (dCERS) technology by single-molecule counting in the journal *Nature*. The report laid an important foundation for the widespread application of SERS. dCERS can realize reproducible, ultrasensitive, and quantitative detection of small molecules, completely changing the methods of small molecule analysis and disrupting the application of traditional technologies. dCERS technology has the ability to produce and prepare large-scale products with convenient detection methods, low costs, and easy calibration, greatly improving the detection efficiency of small molecules. dCERS has recently been used for the pharmacokinetic detection of bioorthogonal drugs in live animals [54] (Figure 2). Bioorthogonal drugs have cyanide, alkyne, and deuterium groups in the drug molecules and can generate Raman signals in the biologically silent window range from 1800 to 2800 cm^−1^, effectively avoiding the signal mixing problem in the Raman spectral fingerprint region (500–1800 cm^−1^) of complex biomolecule systems and improving the reliability and sensitivity of drug molecule signal reading. In addition, the combination of a one-step serum pretreatment scheme reduces the competitive effect of large molecules, such as proteins, in serum on the surface hotspot area of SERS nanoparticles, further improving the sensitivity and reliability of the detection of drug molecules. The pharmacokinetic curve of rats obtained by dCERS is consistent with the experimental verification results of liquid chromatography–tandem mass spectrometry and relevant literature reports, further demonstrating the accuracy and reliability of dCERS in pharmacokinetic monitoring.

dCERS, while offering significant advancements in the field of molecular detection, particularly at ultra-low concentrations, still faces limitations. The primary constraints include the challenge of signal heterogeneity and poor reproducibility at low analyte concentrations, which have been longstanding issues in SERS. Additionally, spectral interference in complex biofluids can limit the broad application of SERS for pharmacokinetics, especially in live animals. Despite these limitations, dCERS holds promise for diverse applications in fundamental studies and clinical tests of bioorthogonal drug molecules due to its sensitivity, controllable accuracy, minimal background interference, and facile pretreatment and measurement.

### 3.2. Enrichment–Detection Systems

Enrichment–detection all-in-one systems can concentrate target analytes, such as drugs or their metabolites, from complex biological matrices, leading to increased sensitivity and selectivity during detection. This enrichment step can significantly improve the LOD compared to colloidal nanoparticles alone. By integrating the enrichment and sensing steps into one platform, the need for separate sample preparation and enrichment processes is eliminated. This can lead to faster analysis times and simplified workflows, which is particularly beneficial for POCT and high-throughput screening.

**Figure 3:**
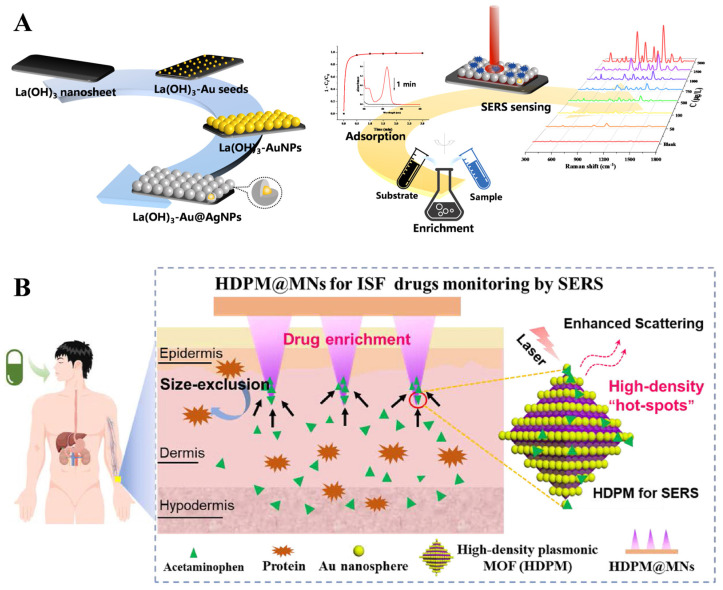
Illustration of (**A**) Ln(OH)_3_-Au@AgNP SERS substrates for enrichment–detection of purine components; adapted from Ref. [40], Copyright© 2023, American Chemical Society, reproduced with permission. (**B**) High-density plasmonic MOF hydrogel-coated SERS microneedles for TDM by SERS; adapted from Ref. [66], Copyright© 2023, American Chemical Society, reproduced with permission.

Solid-phase extraction platforms can provide more consistent and reproducible SERS signals compared to colloidal nanoparticles, which can suffer from variability in the distribution of hotspots and aggregation state. Novel materials enabling rapid adsorption and enrichment can greatly improve the analytical efficiency of SERS in TDM. Lai et al. [40] introduced a rare earth hydroxide to prepare Ln(OH)_3_-Au@AgNPs SERS substrates that could significantly improve the enrichment rate, and the all-in-one enrichment–SERS detection of purine components (mercaptopurine, thioguanine, adenine, and purine) could be completed in 1 min (Figure 3A). The LODs of mercaptopurine and adenine were 6.0 and 0.76 μg/L, with corresponding recoveries of 90.9–100 and 84.2–101%, respectively. Zhan et al. [34] introduced MOF to prepare a Au/Ag@ZIF-8 substrate with excellent solid-phase extraction capability and applied it for label-free SERS monitoring of tacrolimus in human serum. The Au/Ag@ZIF-8 exhibited high stability and molecular sieving effects, realizing sensitive analysis of tacrolimus in the range of 10^−5^–10^−11^ mol/L, with an LOD of 6.4 ng/L. The recovery of tacrolimus in spiked serum samples ranged from 92.0% to 105%, with an RSD of less than 8%.

The coffee ring enrichment effect has also been used for SERS in TDM [55]. The coffee ring effect can naturally enrich analytes at the edge of the coffee ring, and this unique sample preparation method can be achieved without external force, with simple operation, and with minimal sample preparation procedures. This enrichment effect is particularly suitable for use in combination with SERS, greatly improving sensitivity during TDM [55,65]. Zhu et al. [55] developed a coffee-ring-effect-based SERS method that can distinguish clozapine and its metabolites in urine. Silver colloids were dropped on a thin layer chromatography plate for coffee ring enrichment and SERS detection of concentrated clozapine and its metabolites, with a linear test range of 0.5–50 mg/L.

Skin interstitial fluid (ISF)-based microneedle sensing has recently shown broad prospects in minimally invasive and painless diagnosis of diseases [66]. The microneedle tips decorated with plasmonic nanoparticles or enrichment materials can be used for SERS tracking of drug or treatment effects. By adding Au@Ag NPs and hydrogels onto microneedles as SERS sensor, Li et al. [67] realized sensitive and quantitative detection of methylene blue (MB) and mitoxantrone (MTO) in the dermal interstitial fluid of mice within 10 min. The proposed method also showed pharmacokinetic differences between ISF and blood, with the MTO concentration in blood being 10^2^–10^3^ times higher than in ISF. Li et al. [56] developed high-density plasmonic MOF hydrogel-coated SERS microneedles with unique structures characterized by large specific surface areas, specific pore structures, and high-density Au. This design enables selective enrichment and real-time, nondestructive monitoring of acetaminophen levels in dermal interstitial fluid (Figure 3B). The quantitative range of acetaminophen was 1 to 100 μmol/L, with an LOD of 0.45 μmol/L. Additionally, the microneedles demonstrated adequate hardness and flexibility, ensuring minimal damage to dermal tissue during penetration. It can be utilized for pharmacokinetic monitoring of acetaminophen in rats and for adjusting dosage regimens.

In enrichment–SERS detection of TDM, media-modified plasmonic SERS substrates face several challenges: nonspecific fouling by proteins in complex biological fluids, which hampers sensitivity; competitive adsorption with plasma components, reducing assay specificity; and signal interference from non-target molecules. Additionally, these substrates require complex sample pretreatment and are prone to brittleness.

### 3.3. Microfluidics SERS

**Figure 4:**
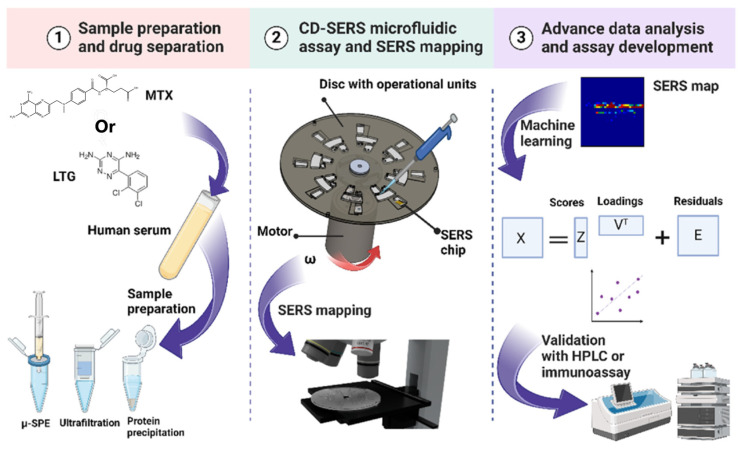
Illustration of solid-phase extraction coupled to automated centrifugal microfluidic SERS for TDM; adapted from Ref. [57], copyright© 2024, Elsevier B.V., reproduced with permission.

Microfluidic technology provides an efficient, sensitive, portable, and cost-effective detection platform for TDM by SERS [68]. A microfluidic platform is a miniaturized lab on a centimeter-scale chip that makes SERS detection more portable and easier to integrate with rapid testing devices. The combination of microfluidics technology and SERS can effectively improve the sensitivity and selectivity of detection. This technology can achieve precise detection of trace drugs and their metabolites, which is crucial for early cancer screening and diagnosis [69]. Microfluidics can achieve rapid detection and accelerated analysis and can accelerate the process of TDM, which is particularly important in clinical environments that require rapid responses. Molecular and cellular analyses can be performed in microchannels on microfluidic chips, thus reducing sample consumption, which is particularly important for precious samples. Wang et al. [70] presented a particle-based PMMS in combination with a two-dimensional Ag SERS substrate device that could realize on-chip separation, identification, and SERS quantification of drug mixtures. An automatic microfluidic control system can control the sample and flow in the device precisely, followed by in-situ SERS detection of the substrate. A hypoxanthine and adenine mixture was quantified, with LODs of 59.0 nmol/L and 22.1 nmol/L for hypoxanthine and adenine, respectively. Multi-channel microfluidic chips enable high-throughput SERS detection, addressing the issue of irregular signal fluctuations and significantly enhancing the reliability of SERS detection. Boisen et al. [57] developed a miniaturized SPE coupled to automated centrifugal microfluidics with incorporated SERS substrates for the analysis of methotrexate and lamotrigine in serum (Figure 4). Firstly, the miniaturized SPE method was used for sample preparation and separation of drugs from human serum. Then, microfluidic discs integrated with SERS substrates loaded with the samples were wetted and mapped accurately and simultaneously by centrifugal rotation. Finally, PLSR was used for big data analysis and quantification of the drugs. The LOD and LOQ of methotrexate were 2.90 and 8.92 μmol/L, and those of lamotrigine were 10.76 and 32.29 μmol/L, respectively.

Microfluidic technology combined with SERS for TDM offers enhanced sensitivity, rapid analysis, and reduced sample consumption. It enables integrated sample processing and detection, making it a powerful tool for rapid on-site diagnostics. Despite these advantages, microfluidic SERS for TDM has several limitations that hinder its broader application. The primary disadvantages include the difficulty in achieving high reproducibility and sensitivity due to the complex fabrication of SERS-active substrates within microchannels. The system’s performance can be compromised by nonspecific adsorption and variability in nanoparticle aggregation, which affects the consistency of SERS signals. Additionally, the integration of bulky external Raman spectrometers with microfluidic devices poses challenges for portability and miniaturization. The high cost of materials and the technical expertise required for device fabrication and operation also limit accessibility. Furthermore, the potential for interference from microchip materials and complex samples in the Raman signal spectrum can reduce detection accuracy. Lastly, the disposal and environmental impact of using nanoparticles as SERS substrates are growing concerns that need to be addressed for sustainable development and use of this technology. Overcoming these challenges is crucial for the advancement of the use of microfluidic SERS in TDM and its transition from research to real-world applications.

### 3.4. Tandem Instrument Technologies

Combining SERS with other instruments in tandem is another solution for improving analytical performance in TDM. The advantages of these tandem instrument technology methods lie in improving the sensitivity and resolution of analysis, expanding the analysis range, providing more structural information, increasing the automation level of detection, reducing detection costs, enhancing patient participation, improving patient efficacy, and enhancing data processing capabilities. Currently, technologies that are combined with SERS include liquid chromatography [58,59], mass spectrometry [60,71], and electrochemistry [61,62].

**Figure 5:**
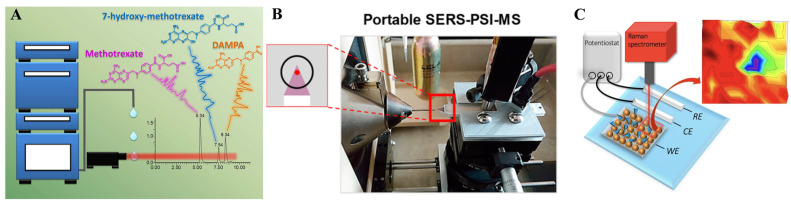
Illustration of (**A**) liquid chromatography coupled with SERS method, adapted from Ref. [58], copyright© 2017, American Chemical Society, reproduced with permission; (**B**) portable SERS-PSI-MS for drug identification, adapted from Ref. [71], copyright© 2020, American Chemical Society, reproduced with permission; and (**C**) electrochemically assisted SERS mapping of metabolites, adapted from Ref. [61], copyright© 2020, American Chemical Society, reproduced with permission.

Liquid chromatography (LC) with good separation capability can separate multiple components over time. Chromatography coupled with SERS can enhance the sensitivity and selectivity of TDM, simplify sample pretreatment, expand application areas, and achieve automation and high-throughput detection. In combination with Raman spectroscopy and a proper SERS substrate, LC-SERS can realize quantitative online multi-component analysis of drugs and their major metabolites with pure and interference-free signals [58,59]. Subaihi et al. [58] developed a method based on liquid chromatography coupled with SERS for fast separation, identification, and determination of eluted drugs (Figure 5A). The method can realize real-time high-throughput separation and detection of methotrexate and its metabolites in spiked human urine, with LODs of 2.36, 1.84, and 3.26 μmol/L. Nguyen et al. [59] presented a liquid chromatography–sheath-flow SERS method that can realize online separation and quantification of mixtures of glucose 1-phosphate, glucose 6-phosphate, and fructose 6-phosphate in cell culture media.

Burr et al. [71] integrated Raman spectroscopy and paper spray ionization–mass spectrometry (PSI-MS) on a single-instrument platform. It improved the sensitivity of detection of drug molecules based on a AuNP-embedded paper swab with a plasmonic electromagnetic enhancement effect (Figure 5B). The LODs of both PSI-MS and SERS were on a scale of 1–100 ng. The integrated SERS-PSI-MS system exhibited a high accuracy of 99.8% in chemical identification in a blind study consisting of 500 samples. Similarly, Erkok et al. [60] developed bifunctional Au/Ag nanostars-loaded paper substrates for SERS and paper spray mass spectrometry analysis of a mixture of fentanyl and fentanyl analogs. The LODs for fentanyl were 34 μg/mL (SERS) and 0.32 μg/mL (PS-MS).

Electrochemistry has also been coupled with SERS for electrochemically assisted separation and enrichment of charged drug molecules, which could shorten analytical time and improve time efficiency. Electrochemical technology has controllable electrode potential, and electric-driven electrochemical enrichment technology enhances sensitivity and selectivity, which is crucial for monitoring trace drugs and their metabolites. Lin et al. [61] employed a negative potential on the conductive AuNPs deposited on indium tin oxide glass substrates, and the Raman intensity of two metabolites of azathioprine (6-thioguanine nucleotides and 6-methylmercaptopurine) significantly increased (Figure 5C). The results were due to the charge transfer effect and Au–S bond formation. The electrochemical–SERS technique can realize 2D mapping for metabolite detection on substrates, with a standard deviation of less than 10%. Quantitative analysis exhibited LODs of 10 nmol/L and 100 nmol/L for 6-thioguanine nucleotides and 6-methylmercaptopurine, respectively. Göksel et al. [62] proposed that an electrochemically assisted SERS procedure for the detection of methotrexate in serum could be conducted in 30 min using gold-coated nanopillar SERS substrates through electrostatic attraction. The linear range was 0.43–2.0 μmol/L and the LOD was 0.13 μmol/L, both of which were clinically relevant levels.

The advantages of the combined instrument method are evident in terms of enhanced SERS sensing performance for TDM. However, the instruments require additional costs for operation and maintenance. The interface between the instruments and the Raman spectrometer depends on the specialized design and expertise of researchers. The stability of the system during long-term operation is another concern. Moreover, the memory effect of plasmonic nanoparticles makes elution difficult, leading to significant interference.

### 3.5. Machine Learning SERS

SERS is a powerful analytical technique that provides molecular fingerprint information with near-single-molecule sensitivity. However, analysis of SERS spectral data is often hindered by their high dimensionality and complexity. Machine learning (ML) has emerged as an essential tool in this context, enabling the extraction of meaningful features from SERS spectra and facilitating rapid, accurate analysis. ML algorithms, such as principal component analysis (PCA), artificial neural networks (ANNs), and support vector machines (SVMs), have been widely employed to enhance the recognition of characteristic peaks, extract effective information, and accelerate data processing in SERS. These techniques have been particularly valuable in distinguishing similar SERS spectra and minimizing interference caused by biological liquids, thereby broadening the applicability of SERS. The integration of ML with SERS not only enhances the accuracy and sensitivity of detection but also paves the way for the development of standardized and benchmarked SERS biosensing platforms, which are crucial for clinical and field-deployable applications.

**Figure 6:**
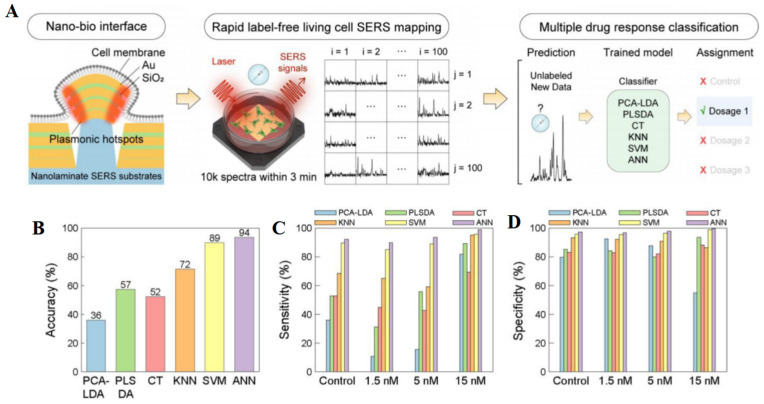
Illustration of (**A**) in-situ SERS measurements and ML classification of cellular drug responses, (**B**) classification accuracy plot, (**C**) sensitivity, and (**D**) specificity of six ML methods for classifying cellular drug responses. Adapted from Ref. [76], Copyright© 2022, American Chemical Society, reproduced with permission.

ML has significant advantages in quantitative SERS analysis. The data generated by SERS are multivariate, and ML algorithms are adept at identifying patterns and trends in complex spectral data for prediction or decision-making. ML algorithms can also process large amounts of SERS data quickly [57]. Through automated data analysis, ML can reduce the impact of human factors on experimental results, thus improving the objectivity and reproducibility of results [72]. ML algorithms can enhance the specificity and sensitivity of SERS, enabling it to more accurately detect and distinguish targets [73]. ML models can learn and generalize to new datasets through training, which means that once the model is trained and validated, it can be used to analyze new SERS data without the need for time-consuming experiments. Diao et al. [74] developed a method that integrates ML algorithms with SERS for quantitative analysis of unlabeled exosomes, enabling precise cancer diagnosis and real-time monitoring of drug efficacy. A three-dimensional AuNP nanofilm rich in “hotspots” was used as a SERS substrate to enhance detection sensitivity and repeatability. The PCA-LDA approach was employed to reduce spectral data dimensionality and boost model classification capabilities. Our ML pipeline included Savitzky–Golay for filtering, airPLS for background signal removal, and Min–Max for spectral normalization, followed by PCA and LDA for data dimension reduction and classification. The model was trained and validated using a random split of 80% and 20% of the data, achieving a 91.1% accuracy in identifying exosomes from various cell lines. SERS spectra changes over time allowed for the dynamic monitoring of chemotherapy drug effects on cancer cells. This study highlights the potential of ML-SERS in biomedical diagnostics, demonstrating accurate discrimination between normal and cancerous exosomes and the monitoring of drug treatment responses. It should be pointed out that the applicability of different ML algorithms varies. Yang et al. [63] developed a magnetic Fe_3_O_4_@COF@Ag SERS substrate combined with ML algorithms for the detection of three quinolone antibiotics (ciprofloxacin, norfloxacin, and levofloxacin). They used different ML algorithms (PCA-k-NN, PCA-SVM, and PCA-Decision Tree) to build a multi-classification model. The performance of the k-NN algorithm in classification tasks is greatly influenced by the choice of the k value. According to the results, the average accuracy of the k-NN algorithm after PCA preprocessing can reach 88% with k values ranging from 3 to 9. However, the decision boundary of k-NN is too precise, especially in NFX (norfloxacin) classification, which leads to overfitting of the model and requires high-cost adjustment of the k value. The SVM algorithm is particularly suitable for small datasets [75], and its decision boundary leaves sufficient spacing between the three antibiotics. According to the results, PCA-SVM (kernel function RBF) can accurately classify the SERS spectra of three antibiotics with accuracy, recall, and precision of 100%. In addition, the search results also showed that PCA-SVM achieved an accuracy of 97.6% in the external validation set. This indicates that PCA-SVM achieves excellent performance in classification tasks. The decision boundary of the decision tree is very close to the training sample, with a small interval; thus, it is not a good choice. According to the results, the decision tree’s recognition accuracy for NFX in the training set is only 33%, which is the worst performance. Similarly, Nam et al. [76] prepared nanolaminate plasmonic substrates for high-throughput living cell SERS measurements and selected ANN classification of cellular drug responses (Figure 6). They found that an ANN was very effective at classifying how cells respond to different drugs, with a 94% accuracy rate. The researchers used special SERS tools that can quickly collect a lot of detailed data from living cells, which helped the ANN work well. The ANN was better at this task than other methods like PCA-LDA, PLSDA, CT, k-NN, and SVM because it could find hidden patterns in the data that the other methods missed.

Although ML promotes SERS for TDM, mostly when it comes to the analysis of data, the results indicate that the issues raised earlier cannot be effectively resolved, for example, the properties of enhancement substrate, matrix interference, and inherently weak signals of some drugs. As a tool for adding icing on the cake, ML-enabled SERS for complex TDM also faces several challenges: (i) Data quality and quantity issues: Machine learning models require a large amount of data for training. Obtaining a sufficient number of high-quality, labeled SERS-TDM samples can be challenging. In the case of biological samples, differentiated background contributions from different undesirable components can interfere with the signal and require ML algorithms to extract useful information. (ii) Signal fluctuations and noise. (iii) The applicability of deep learning networks: It is believed that the more complex the network structure, the better the model performance; however, complex deep learning networks may not be suitable for feature mining from small sample data. (iv) Model generalization ability: ML models trained on specific datasets may not generalize well to new, unseen samples, especially if there are significant differences in sample composition.

## 4. Conclusions and Perspectives

The integration of SERS in TDM has demonstrated significant potential for revolutionizing the precision of personalized medicine. This review highlights the advancements in SERS substrates and their applications in TDM, showcasing the high sensitivity, specificity, and low sample consumption of SERS. The innovative approaches, such as digital colloid-enhanced Raman spectroscopy, enrichment–detection, microfluidic SERS, instrument tandem technology, and machine learning-enabled SERS, have addressed several challenges associated with traditional TDM methods. These include improving selectivity, overcoming matrix interference, and enhancing the signal reproducibility of SERS substrates. The collective progress in substrate design, analytical method development, and data analysis has positioned SERS as a promising tool for TDM, with the potential to transform drug monitoring through its rapid, accurate, and cost-effective analysis.

Looking forward, ongoing research in SERS for TDM is expected to further refine substrate design, reduce costs, and develop user-friendly data analysis tools. The development of robust standardization protocols will be crucial for enhancing the technique’s utility and acceptance in clinical settings. The synergy between SERS and emerging technologies such as microfluidics, nanotechnology, and artificial intelligence offers a plethora of opportunities for innovative solutions in TDM. Future work should focus on translating these laboratory advancements into real-world clinical applications, ensuring the techniques are user-friendly and the devices are portable for POCT. Additionally, the exploration of SERS in monitoring drug resistance, personalized dosing regimens, and real-time pharmacokinetic studies will be of paramount importance. As the field of SERS advances, the synergy between material scientists, clinicians, and data analysts will play a crucial role in maximizing SERS’s impact on TDM, thereby enhancing patient outcomes.

## Figures and Tables

**Table 1:** A summary of SERS-based detection of different therapeutic drugs.

Drug Molecules	Sample	Substrate	Linear Range (mol/L)	LOD (mol/L)	Recovery (%)	Ref.
6-Mercaptopurine		AuNPs	(5–20) × 10^−6^	1.0 × 10^−6^		[22]
6-Mercaptopurine		AuNPs	(1–15) × 10^−6^	0.1 × 10^−6^		[23]
Methotrexate		Ag colloid	1 × 10^−16^–1 × 10^−6^	1 × 10^−16^	96.4–104.3	[24]
Tacrolimus	serum	Au/Ag@ZIF-8	10^−5^–10^−11^	6.4 ng/L	92–105	[34]
Benzodiazepines	serum	Fe_3_O_4_@PDA@Au		50 ng/mL		[36]
Carbamazepine, clozapine	serum	Fe_3_O_4_@SiO_2_@MIL-101(Fe)	0.1–100 mg/L	0.072, 0.12 mg/L	94.0–105.0	[37]
Ritonavir, ibrutinib	serum	Ti_3_C_2_T_x_/Ag NCs	10^−1^–10^−6^ g/L, 10^−1^–10^−5^ g/L	5.62 × 10^−7^, 1.1 × 10^−6^ g/L	>90.0	[39]
Mercaptopurine	tablets	La(OH)_3_-Au@AgNPs	0.05–5.0 mg/L	6.0 μg/L	90.9–100.0	[40]
Tetracycline		aptamer-modified AAO	1 pg/L–1 μg/L	1 pg/L		[43]
Caspase-3	apoptotic cells	TiO_2_/Au NTAs	1.0 ng/L–10 μg/L	0.25 ng/L	91.0–109.0	[46]
Doxorubicin	serum	Au@SiNPs	1 × 10^−9^ –1 × 10^−6^	2.0 × 10^−8^		[48]
Urotropine, 2,5-dimethylpyrazine, pyrazinamide, pyrazine	human plasma	Ag/PNIP-LAP	0.070–5.00 mg/L	1.11–53.1 μg/L	84.3–106.0, 81.8–99.7, 84.4–116.8	[49]
Aminophylline	serum	Ag@PNIPAM	1–1.1 × 10^2^ mg/L	0.61 mg/L	3.0–101.8	[50]
Phenytoin	PBS	Au poly-SERS films	10–20 mg/L	1.8 mg/L		[51]
Methotrexate	serum	AgNPs chip	(5–150) × 10^−6^	2.1 × 10^−6^		[52]
Acetaminophen	sweat	Au nanosphere cone array	(0.5–100) × 10^−6^	0.13 ×10^−6^		[53]
Erlotinib	rat serum	citrate–Ag colloids	10^−8^–10^−5^ g/L	10^−7^ g/L		[54]
Clozapine	urine	TLC-coupled CRE-SERS	0.5–50 mg/L	0.1 mg/L	86.9–119.6	[55]
Acetaminophen	interstitial fluid	HDPM@MNs	(1–100) × 10^−6^	0.45 × 10^−6^		[56]
Methotrexate, lamotrigine	serum	Ag NPs		(2.90, 10.7) × 10^−6^		[57]
Methotrexate, 7-hydroxy methotrexate, 2,4-diamino-N(10)-methylpteroic acid	human urine	silver colloid		(2.36, 1.84, 3.26) × 10^−6^		[58]
Phosphorylated carbohydrates		silver	(0.25–20) × 10^−6^			[59]
Fentanyl		Au/Ag nanostars	2.5–0.1 mg/L	34 mg/L		[60]
6-Thioguanine nucleotides, 6-Methylmercaptopurine		AuNPs	(0.02–2) × 10^−6^ (1–20) × 10^−6^	(10, 100) × 10^−9^		[61]
Methotrexate	serum	Au-Capped NP	(1.81–5) × 10^−6^	0.55 × 10^−6^		[62]
Ciprofloxacin, Norfloxacin, Levofloxacin		Fe_3_O_4_@COF@Ag	1 × 10^−8^–1 × 10^−4^ 1 × 10^−7.5^–1 × 10^−4^	5.61 × 10^−9^ 1.44 × 10^−8^ 1.56 × 10^−8^	95.9–103.1 96.3–109.2 91.4–108.1	[63]
